# Evaluation of Interventions to Reduce Firefighter Exposures

**DOI:** 10.1097/JOM.0000000000001815

**Published:** 2020-04

**Authors:** Jefferey L. Burgess, Christiane Hoppe-Jones, Stephanie C. Griffin, Jin J. Zhou, John J. Gulotta, Darin D. Wallentine, Paul K. Moore, Eric A. Valliere, Sasha R. Weller, Shawn C. Beitel, Leanne M. Flahr, Sally R. Littau, Devi Dearmon-Moore, Jing Zhai, Alesia M. Jung, Fernanda Garavito, Shane A. Snyder

**Affiliations:** Department of Community, Environment and Policy, Mel and Enid Zuckerman College of Public Health, University of Arizona, Tucson, Arizona; Department of Chemical and Environmental Engineering, College of Engineering, University of Arizona, Tucson, Arizona; Department of Community, Environment and Policy, Mel and Enid Zuckerman College of Public Health, University of Arizona, Tucson, Arizona; Department of Epidemiology and Biostatistics, Mel and Enid Zuckerman College of Public Health, University of Arizona, Tucson, Arizona; Tucson Fire Department, Tucson, Arizona; Tucson Fire Department, Tucson, Arizona; Tucson Fire Department, Tucson, Arizona; Scottsdale Fire Department, Scottsdale, Arizona; Scottsdale Fire Department, Scottsdale, Arizona; Department of Chemical and Environmental Engineering, College of Engineering, University of Arizona, Tucson, Arizona; Department of Chemical and Environmental Engineering, College of Engineering, University of Arizona, Tucson, Arizona; Department of Community, Environment and Policy, Mel and Enid Zuckerman College of Public Health, University of Arizona, Tucson, Arizona; Department of Community, Environment and Policy, Mel and Enid Zuckerman College of Public Health, University of Arizona, Tucson, Arizona; Department of Epidemiology and Biostatistics, Mel and Enid Zuckerman College of Public Health, University of Arizona, Tucson, Arizona; Department of Epidemiology and Biostatistics, Mel and Enid Zuckerman College of Public Health, University of Arizona, Tucson, Arizona; Department of Community, Environment and Policy, Mel and Enid Zuckerman College of Public Health, University of Arizona, Tucson, Arizona; Department of Chemical and Environmental Engineering, College of Engineering, University of Arizona, Tucson, Arizona

**Keywords:** exposure reduction, firefighter, intervention, polycyclic aromatic hydrocarbon, sauna, SCBA, wash down

## Abstract

**Objective:**

Evaluate the effectiveness of firefighter exposure reduction interventions.

**Methods:**

Fireground interventions included use of self-contained breathing apparatus by engineers, entry team wash down, contaminated equipment isolation, and personnel showering and washing of gear upon return to station. Urinary polycyclic aromatic hydrocarbon metabolites (PAH-OHs) were measured after structural fire responses before and after intervention implementation. Separately, infrared sauna use following live-fire training was compared to standard postfire care in a randomized trial.

**Results:**

The fireground interventions significantly reduced mean total urinary postfire PAH-OHs in engineers (−40.4%, 95%CI −63.9%, −2.3%) and firefighters (−36.2%, 95%CI −56.7%, −6.0%) but not captains (−11.3% 95%CI −39.4%, 29.9%). Sauna treatment non-significantly reduced total mean PAH-OHs by −43.5% (95%CI −68.8%, 2.2%).

**Conclusions:**

The selected fireground interventions reduced urinary PAH-OHs in engineers and firefighters. Further evaluation of infrared sauna treatment is needed.

## INTRODUCTION

Firefighters are at higher risk for multiple cancers than the general population,^1,2^ with cancer incidence and mortality increasing with time spent at the fire scene and number of fire runs for lung cancer and leukemia, respectively.^3^ Exposure to multiple known and suspected human carcinogens, including some polycyclic aromatic hydrocarbons (PAHs), benzene, and formaldehyde, have been documented in products of combustion at the fireground.^4,5^ The measurement of hydroxylated metabolites of PAHs in urine (PAH-OHs) has been used extensively as a biomarker of firefighter exposure^6–14^ and reflects exposure from inhalation, skin exposure, and ingestion. Many urinary PAH-OHs have biological half-lives of the order of several hours or less and generally serve as a marker of short-term exposures.^15^

Fire departments are increasingly putting into practice strategies to reduce or mitigate exposure to carcinogens, focusing on both inhalation and dermal exposure routes. These include increased use of self-contained breathing apparatus (SCBA) during overhaul, more rapid dermal decontamination at the fire scene, postfire personal protective equipment (PPE) decontamination to reduce firefighters’ exposure to combustion products via off-gassing from contaminated gear and dermal transfer, taking showers and changing clothes as soon as possible upon return to the station, and washing turnout gear after each fire.^16–18^ In addition, some fire departments are providing saunas for use after returning to the station postfire incident.

Only limited information is available on the effectiveness of fire department interventions on reducing the concentration of toxicant biomarkers in the body of firefighters, and uncertainty over the efficacy of these practices limits their implementation.^17–19^ In addition, even well-intended interventions may have adverse effects. For example, use of air purifying respirators during overhaul led to poorer respiratory outcomes than use of no respiratory protection at all, resulting in the recommendation to use SCBA during overhaul.^5^ The purpose of the current study was to evaluate the effectiveness of specific interventions chosen by the fire service, including fireground interventions put in place by the Tucson Fire Department (TFD) for structural fire responses and the use of postexposure infrared saunas by the Scottsdale Fire Department (SFD).

## METHODS

The fireground exposure reduction intervention study implemented by the TFD was part of a larger cancer prevention research project approved by the UA IRB, Protocol # 1509137073. The sauna intervention study implemented by the SFD was separately approved by the UA IRB. All subjects provided informed consent prior to entry into the study.

### Fireground Intervention Study

The fireground intervention study included baseline, pre-intervention postexposure, intervention training and post-intervention postexposure components. All TFD firefighters were eligible for inclusion in the study. Participating subjects completed a survey on their occupational and medical history at baseline, when they entered into the study. Biological samples collected at this time included urine, blood, and buccal cells. Collection of baseline samples started in October, 2015 and extended through July, 2018. During the pre- and post-intervention postexposure periods, firefighters were monitored for exposure to products of combustion by collecting urine 2 to 3 hours following cessation of their fire response. TFD predominantly selected residential structural fires for evaluation as this was the most common type of fire event. Industrial fires were excluded from evaluation. The pre-intervention exposure evaluation period began in February, 2016 and extended through January, 2017. The post-intervention period extended from November, 2017 to March, 2019. All TFD personnel were trained on the new interventions from October to November of 2017, with continuing reminders thereafter.

TFD used the results of the pre-intervention urinary PAH-OH analyses to plan multiple exposure reduction interventions to minimize both inhalation and dermal exposure. These included use of SCBA by engineers (“engineers on air”) and fire cause investigators, surface contamination reduction (“wash down”) of turnout gear and SCBA predominantly worn by entry teams by cleaning the gear with soap and water prior to doffing, additional skin decontamination, and segregation of contaminated gear prior to transport and additional cleaning of gear upon return to the station. TFD focused the post-intervention evaluation on fire crews expected to have followed the recommended interventions.

The engineers on air intervention was selected based on the assumption that their exposure was primarily due to a lack of respiratory protection, as they did not participate in interior firefighting and generally did not show evidence of soot deposition on their turnout gear or skin. Prior to the intervention, first-in engineers operating at the pump panel or aerial and/or securing utilities generally operated without an SCBA. The intervention involved the recommendation that, as soon as practical, engineers should don their SCBA and be on positive pressure air while exposed to smoke.

The postfire wash down was selected to reduce self-contamination by soot deposition on turnout gear and SCBA, exposure to off-gassing during and after doffing of gear and contamination of other personnel treating the exposed firefighter and/or subsequently handling the gear. This intervention was chosen based on a previous study demonstrating that two minutes of brushing with soap and water removed a median of 85% of PAHs from the turnout ensemble.^18^ The intervention involved gross external decontamination of turnout gear prior to removing the firefighting ensemble (including SCBA regulator) worn in the hot zone. The firefighters brushed off large debris first and then sprayed each other with water to remove loose particulates. The wash down kit included a bucket with a lid, 2.5′′ to green line reducer, hose, nozzle, brush, and soap (Dawn Ultra Dishwashing Liquid, Procter & Gamble, Cincinnati, OH). The gear was washed for ~ 2 minutes, with water pressure limited to avoid drenching of the gear. After the wash down, the turnout gear was removed prior to reporting to rehabilitation (“rehab”) at the fire scene. Following return to their station, the firefighters were encouraged to shower as soon as possible, ideally within an hour, and put on clean clothing.

The practice of bagging of gear and maintaining a “clean cab” was selected based on the premise that products of combustion should be treated in a similar fashion as any other biohazard. Contaminated hose, tools, SCBAs, or any other contaminated equipment were to be decontaminated on scene and/or transported in a manner as to not contaminate the cab of the truck. Clear plastic bags were carried by each executive captain so that gear could be bagged and easily identified. Fire hose and any other dirty gear were bagged or transported separately from the cab. Upon arrival back at the station, the bags were opened outside the bays and allowed to off-gas before cleaning.

In the station, all contaminated gear was washed in an extractor (UniMac and Wascomat) with turnout manufacture approved mild detergent (ECOLAB Tri-Star Flexylite), using nitrile gloves and eye protection. The outer shells were separated from the inner liners and washed separately with the manufacturer recommended extractor wash cycles for each with parameters determined by the device specifications, calibrated by the vendor. Boots were scrubbed and gloves, helmet pieces and SCBA facepieces were hand washed with warm water. During the intervention TFD also increased the number and size of the station extractors, allowing for more turnouts to be cleaned with each wash cycle.

Two separate surveys, one on-scene and another following return to the station (in-station), were completed using tablet computers during the pre- and post-intervention periods. The on-scene survey was taken during rehab and collected information on the subject’s role during the response, extinguishing agents used, PPE worn, medical symptoms, how long it had been since they were involved in fire suppression prior to this response and how long it had been since their turnout gear had been washed. The in-station survey, completed two to three hours after the end of the fire response, included questions on soot on their gear and/or skin, bagging their gear prior to leaving the fire scene, showering within an hour and cleaning and storage of gear. In addition, questions were asked concerning exposures in the past two weeks not related to their firefighting activities, including smoking, grilling foods, beverages, refueling vehicles, and bicycling.

### Sauna Intervention Study

SFD firefighters scheduled for annual live-fire fire training were eligible for study participation. Exclusion criteria included current smoking (including cigarettes, cigars, and e-cigarettes) and contraindication to ingestion of the core body temperature monitor probe, including impairment of the gag reflex, a swallowing disorder, diseases or disorders of the esophagus, previous gastrointestinal surgery or obstructive disease of the gastrointestinal tract, a low motility disorder of the gastrointestinal tract, a cardiac pacemaker or other implanted electromedical device and undergoing nuclear magnetic resonance or magnetic resonance imaging scanning less than 3 days after swallowing the sensor. Urine was collected for 12 hours prior to the anticipated annual fire training start time and for 12 hours following completion of the live-fire training. The firefighters ingested a temperature probe and wore a core body temperature monitor (CorTemp Data Recorder, HQ, Inc., Palmetto, FL) and a chest heart rate belt (Polar Heart Rate Chest Belt, Polar, Bethpage, NY) during the fire and for 8 hours afterward, with the exception of removal during showering.

The sauna intervention study was conducted over three days in 2018 (May 8th, May 10th, and September 5th) with two crews of three subjects on each of the first two dates and four crews of three subjects each on the final date. The study utilized live fire training evolutions conducted annually for every SFD firefighter as a continuing education requirement. The evolutions were conducted in a fire service training burn building with each burn utilizing one wooden pallet, a 1/4 bale of hay, and 1.44 square meters of oriented strand board. The evolutions simulated a residential structure fire with crews wearing SCBA being deployed interior for the tactical objectives of fire attack and search and rescue. The approximate time operating on the interior was 10 to 15 minutes for each evolution. The subjects participated in two evolutions with a 30-minute break in between to rehydrate, refill SCBA bottles, and a quick critique of the first evolution. No decontamination efforts were conducted during the break. The second evolution was conducted with the same burn materials and a similar scenario, except crews alternated assignments for deployment and arrival order. After completion of the second scenario, the crews went to full rehab and conducted their standard decontamination protocols including cleaning their face, neck, hands, and arms with wipes soaked in a dish soap and water solution. Water and electrolytes were provided for rehydration and mister fans were utilized to facilitate cooling. All PPE was taken out of service for cleaning. During rehab, three of the six firefighters were randomly selected for sauna treatment. All firefighters showered after rehab, usually within 20 to 30 minutes of completion of the second evolution. The firefighters not assigned to the sauna treatment left the training yard after showering. Both groups had been instructed not to eat grilled meat 24 hours before and during the 12-hour postexposure urine collection period.

The firefighters randomly selected for sauna treatment entered the sauna (Dynamic Palermo 3-person FAR Infrared Sauna, Model DYN-6330–01, Dynamic Saunas, Ontario, CA) immediately after showering. The mean time between exiting the burn building at the end of the firefighters’ second evolution and entering the sauna was 42 minutes, with a range of 36 to 47 minutes. The subjects rested in the sauna for 20 minutes at a temperature setting of 49°C (120 °F), wearing standard fire department station physical training clothing (shorts and t-shirt) and sitting on and utilizing clean towels to absorb sweat. The subjects then took an additional shower immediately after exiting the sauna and then left the training yard. The sauna treatment protocol was determined by SFD based on a time interval that would be reasonable when utilized on duty and a temperature which would be acceptable to the firefighters. While in the sauna the subjects drank water *ad libitum* on the first 2 days of testing but did not have access to water on the final day of testing.

### Urine Collection and Analysis

The firefighters were instructed to wash their hands prior to urine collection and to collect each full void using as many urine cups as needed. Samples were stored at 0 to 8°C until they could be transported on ice to the laboratory for processing. For the sauna intervention study, 50 mL 12 hour pre-exposure and postexposure composite urine samples were created by calculating the sum of each full void collected and adding them together to determine the total volume, dividing each time point’s volume by the total volume and multiplying by 50 mL. The volume calculated for each time point was then added to a 50 mL conical tube, centrifuged at 1900 rpm for 10 minutes and the supernatant frozen at −20°C until analyzed. Specific gravity was measured on the 12-hour composite baseline, 2 to 4 hour postexposure, and 12-hour the postfire composite samples by refractometry (Atago “Pocket” Urine Specific Gravity Refractometer, Atago Co., Bellevue, WA).

Urine samples were analyzed for 10 PAH-OHs (1-naphthol, 2-naphthol, 2-fluorenol, 3-fluorenol, 4-fluorenol, 1-phenanthrol, 2-phenanthrol, 3-phenanthrol, 4-phenanthrol, and 1-hydroxypyrene) as previously described.^20^ In short, 3 mL urine samples were spiked with a mix of isotopically labeled PAH-OHs (1-hydroxynaphthalene-*d*7, 9-hydroxyphenanthrene-*d*8, 2-hydroxyphenanthrene-*d*9, 2-hydroxyfluorene-*d*9, and 1-hydroxypyrene-*d*9). After the addition of 10 uL β-glucuronidase from *Helix pomatia* (Sigma Aldrich, Milwaukee, WI) and 5 mL of sodium acetate buffer, the samples were incubated at 37°C for 16 to 18 hours and extracted using Bond Elut Focus SPE cartridges (Agilent Technologies, Santa Clara, CA). After loading and drying of the cartridges, they were eluted with 6 mL dichloromethane. The solvent in the extracts was exchanged to nonane and the samples derivatized with MSTFA. The derivatized extracts were analyzed on a GC-MS 7890A (Agilent Technologies, Santa Clara, CA).

### Statistical Analyses

Non-detectable levels of individual PAH-OHs were replaced by half of their detection limits (175/2 ng/L for naphthols, 100/2 ng/L for fluorenols, 150/2 ng/L for phenanthrols and 200/2 ng/L for 1-hydroxypyrene). Non-detectable PAH-OH sums were replaced by their machine limits (175 ng/L for sum of naphthols, 100 ng/L for sum of fluorenols and 225 ng/L for sum of phenanthrols). All PAH-OH concentrations were log transformed. For fireground interventions multivariable analyses were performed using a linear mixed effects model with random intercept to assess mean differences of log-transformed PAH-OHs, comparing pre- and post-interventions stratified by job types. For the analysis of sauna intervention effects on PAH-OHs, a linear mixed effect model was also adopted. The effect of sauna intervention compared with control treatment on PAH-OHs over time (ie, at baseline, after 2 to 4 hours, after 12 hours) was estimated by adding a “treatment” by “time” interaction. The mean difference of PAH-OH measurements between those with sauna intervention and controls after 12 hours was estimated and the proportion of this difference over the mean PAH-OH measurements after 12 hours with no sauna intervention was reported. Assessment of model fit was performed by the analysis of residuals. All statistical analyses were performed using R version 3.6.0 (https://www.r-project.org). Longitudinal analyses were conducted by the R package “lme4.” Confidence intervals of ratio were assessed by Fieller’s theorem and R package “mratio.”^21^ A two-sided *P*<0.05 was considered statistically significant.

## RESULTS

The participating firefighters from both departments were predominantly non-Hispanic white males (Table 1). The average ages were 38.1 and 38.6 years at baseline for the TFD firefighters providing postexposure urines and SFD firefighters, respectively. Despite randomization, the SFD firefighters in the control group were significantly older than the sauna intervention group. Body mass index averaged 27.9±3.4, 27.8±3.3, and 27.2±3.0 kg/m^2^ in the TFD baseline, pre-intervention and post-intervention groups, respectively. Height and weight information were not available for the SFD firefighters. For TFD subjects, 242 of the 255 total subjects provided a baseline urine sample, 104 provided at least one postexposure pre-intervention urine and 54 provided at least one postexposure post-intervention urine. Thirty-six firefighters provided more than one pre-intervention urine, ranging up to six samples, and eight firefighters provided more than one post-intervention urine, ranging up to three samples. Eleven firefighters provided at least one pre-intervention urine and at least one post-intervention urine. SFD firefighters participated only once in the sauna intervention study. The analysis was limited to the results of urine testing of engineers, firefighters, and captains, as only these groups had sufficient numbers of pre- and post-intervention subjects for statistical comparison.

The TFD firefighters responded to 15 fires in the pre-intervention period and 13 fires in the post-intervention period. The fires in each group were similar, consisting of residential and commercial structure fires. The pre-intervention fires included ten homes, one house and car combination, one apartment, two commercial structures, and one school. Three of the fires were mostly defensive following an interior fire attack. Four fires involved two or more rotations of firefighters returning to the involved structure after rehab to perform additional firefighting functions and overhaul. Average response time for the 13 fires for which this information was complete was 36 minutes. The post-intervention fires included eight homes, two apartments and two commercial structures, including one hotel. One of the fires was mostly defensive following an interior fire attack and one involved two or more rotations of firefighters returning to the involved structure after rehab to perform additional firefighting functions and overhaul. The average response time for the 13 fires was 38 minutes.

**FIGURE 1.**
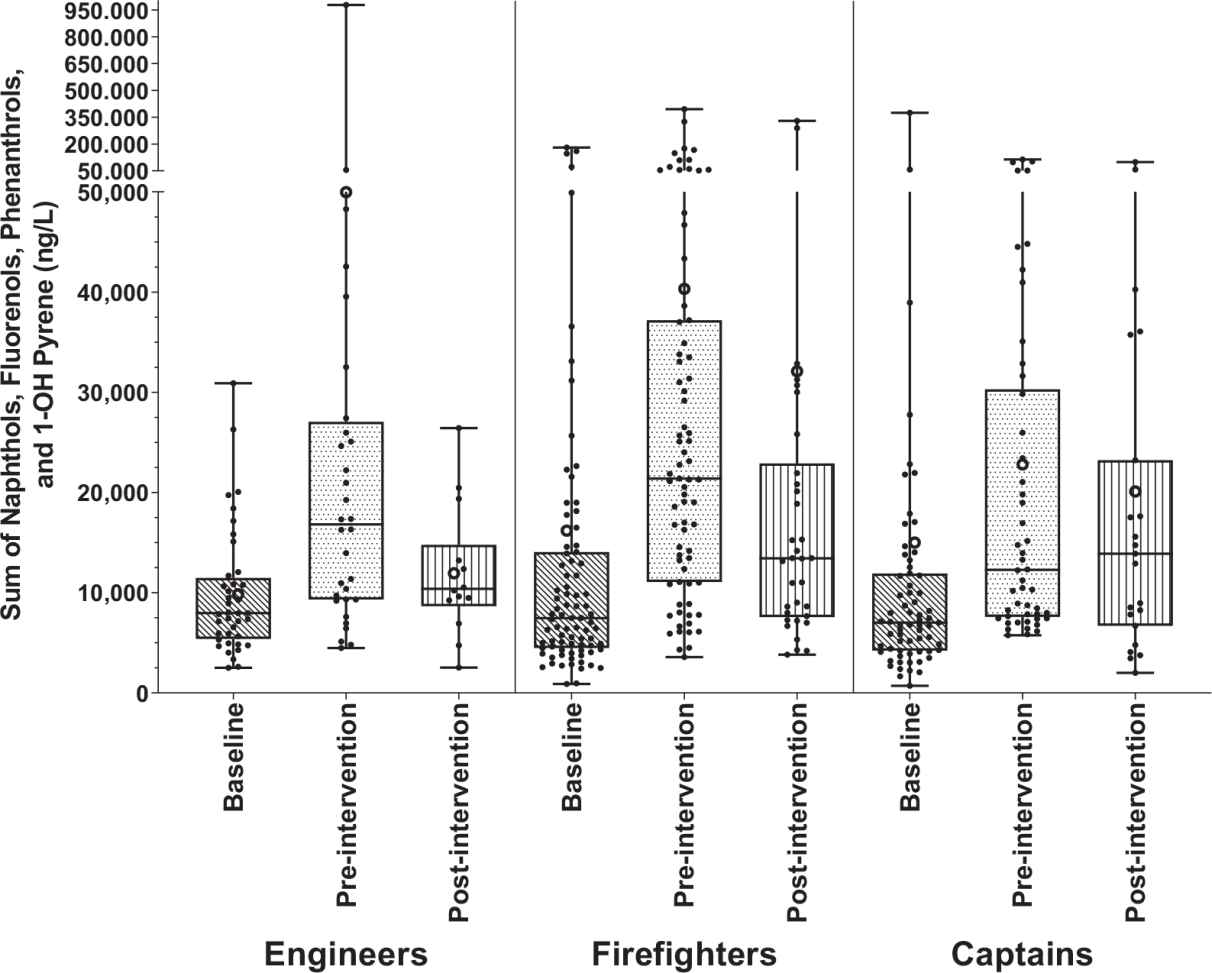
Fireground urinary PAH-OH measurements by job classification (open circle=mean). PAH-OH, polycyclic aromatic hydrocarbon metabolites.

For fireground interventions, engineers showed a statistically significant 40.4% reduction in urinary mean concentration of all naphthol, fluorenol, and phenanthrol metabolites and 1-hydroxypyrene combined (Σ sums) comparing post-intervention to pre-intervention time periods (Table 2). Firefighters showed a significant 36.2% mean reduction in Σ sums, and captains showed a non-significant 11.3% mean reduction. The distribution of urinary PAH-OHs at baseline, pre-intervention, and post-intervention are shown in Figure 1. The statistical significance of reduction in specific isomer groups of PAH-OHs comparing pre- and post-intervention periods varied by fire service activity: sum of naphthols, only firefighters; sum of fluorenols, engineers and firefighters; and sum of phenanthrols, engineers, firefighters, and captains. The results for individual PAH-OHs are listed in Supplementary Table 1, http://links.lww.com/JOM/A707. There was a wide range of urinary PAH-OH concentrations within each group; for example, one engineer in the pre-intervention group had a urinary 1-naphthol measurement (585,300 ng/L, confirmed by reanalysis) more than twice the level of the second highest measurement.

For the pre-intervention phase, 180 on-scene and 120 in-station surveys were completed for individuals that also provided a postexposure urine (Table 3). For the post-intervention phase, 67 on-scene and 60 in-station surveys were completed. These show a 15% increase during the post-intervention period in having clean gear before the response and a smaller increase in various PPE worn during fire attack (range 8% to 13%) and overhaul (range 3% to 6%). In regards to respiratory protection, SCBA use increased 13% during fire attack, which included both interior and exterior attack, and 8% during overhaul. Use of skin wipes/washing with water on-scene and replacing hoods on scene, both practices put in place prior to the study interventions, increased 14% and 2%, respectively. Wash down of turnout gear and SCBA on-scene, both new interventions, increased 58% and 35%, respectively. There was less emphasis on wash down for engineers given that they did not do interior fire response or ventilation. Excluding the 10 engineers with survey responses from the question “washed/rinsed/or replaced the following on scene,” the percentages of subjects responding positively increased to 82% for hoods and 76% for turnout gear and stayed at 75% for SCBA. For all subjects combined (including engineers), bagging dirty gear and storing it outside of the cab increased 28% and 15%, respectively, while there was a 10% decline in both showering and washing/replacing clothes within an hour after the response. All four of these activities were the focus of the new interventions.

**FIGURE 2.**
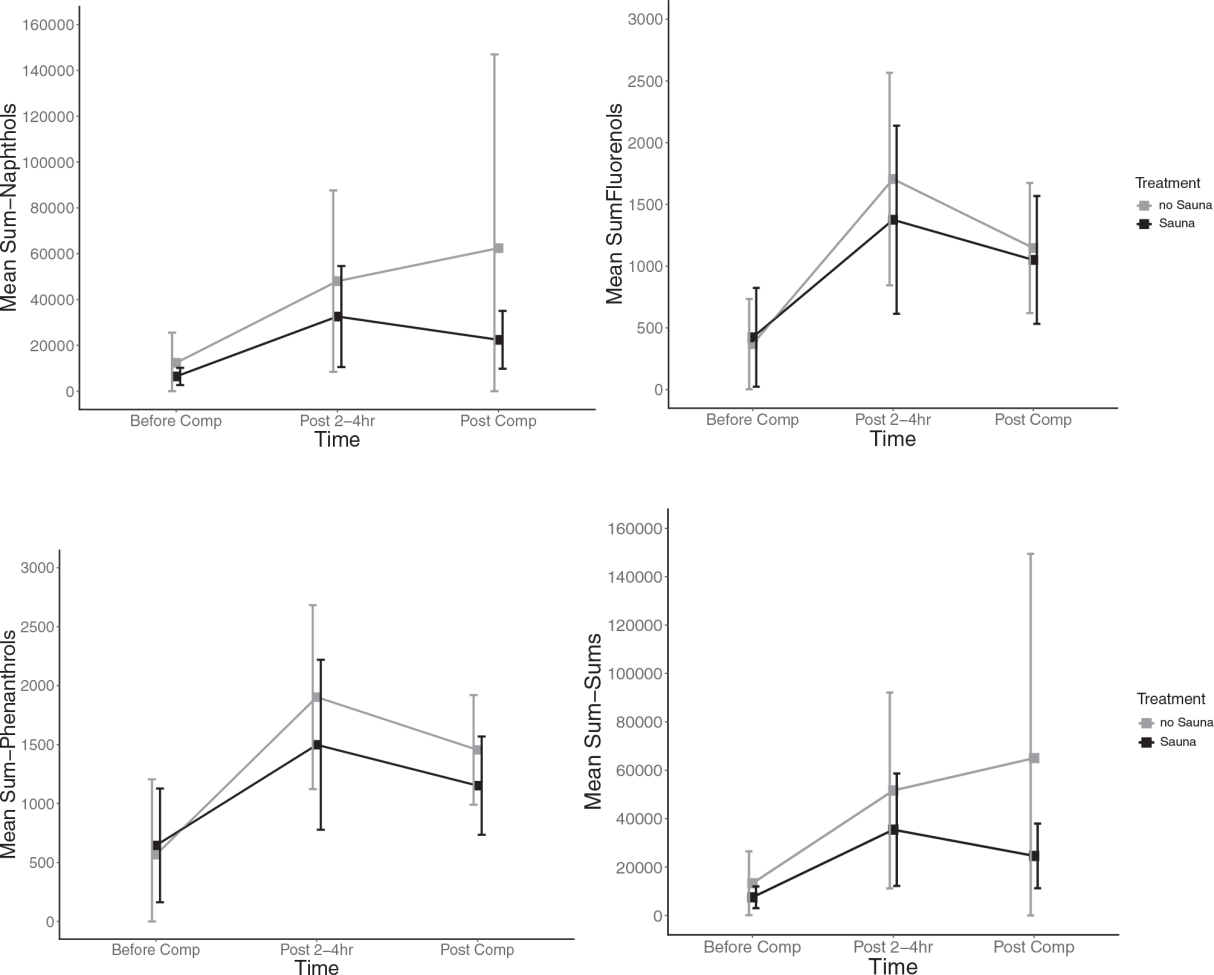
A–D: Mean (SD) of PAH-OHs (ng/L) before and after (2 to 4 hours and 12 hour composite) firefighting by treatment group. PAH-OH, polycyclic aromatic hydrocarbon metabolites.

For the sauna intervention, there was a non-significant 43.5% decrease in the geometric mean PAH-OH Σ sums concentration in the 12-hour postexposure composite urine sample for those firefighters randomized to infrared sauna treatment compared to the controls (Table 2). While also not statistically significant, there were greater reductions in sum of naphthols than sum of fluorenols and sum of phenanthrenes. As with the fireground intervention groups, there was a wide range of urinary PAH-OH concentrations within each group (Supplementary Table 2, http://links.lww.com/JOM/A708). The highest individual urinary PAH-OH measurement was a 2-naphthol level of 295,808 ng/L in a control subject, confirmed by reanalysis. For the 2 to 4 hour postexposure geometric mean urinary PAH-OH concentrations, comparing the and control and sauna treatment groups respectively, there were non-significant reductions in sum of naphthols (36,431±2.2 and 25,982±2.1 ng/L, *P*=0.08), sum of fluorenols (1,367±1.8 and 1,048±1.8 ng/L, *P*=0.39), sum of phenanthrols (1,730±1.6 and 1,359±1.5 ng/L, *P*=0.48) and Σ sums (41,832±2.0 and 29,522±2.0 ng/L, *P*=0.07). The 12-hour composite pre-exposure, 2 to 4 hour postexposure and 12-hour composite postexposure urinary sums are shown graphically in Figure 2A to D. The mean urine specific gravities in the control and sauna treatment groups were 1.012±0.004 and 1.015±0.006 (*P*=0.13) at baseline, 1.014±0.007 and 1.018±0.008 (*P*=0.17) at 2–4hours and 1.012±0.007 and 1.016±0.007 (*P*=0.18) for the 12-hour postexposure composite samples, respectively.

**FIGURE 3.**
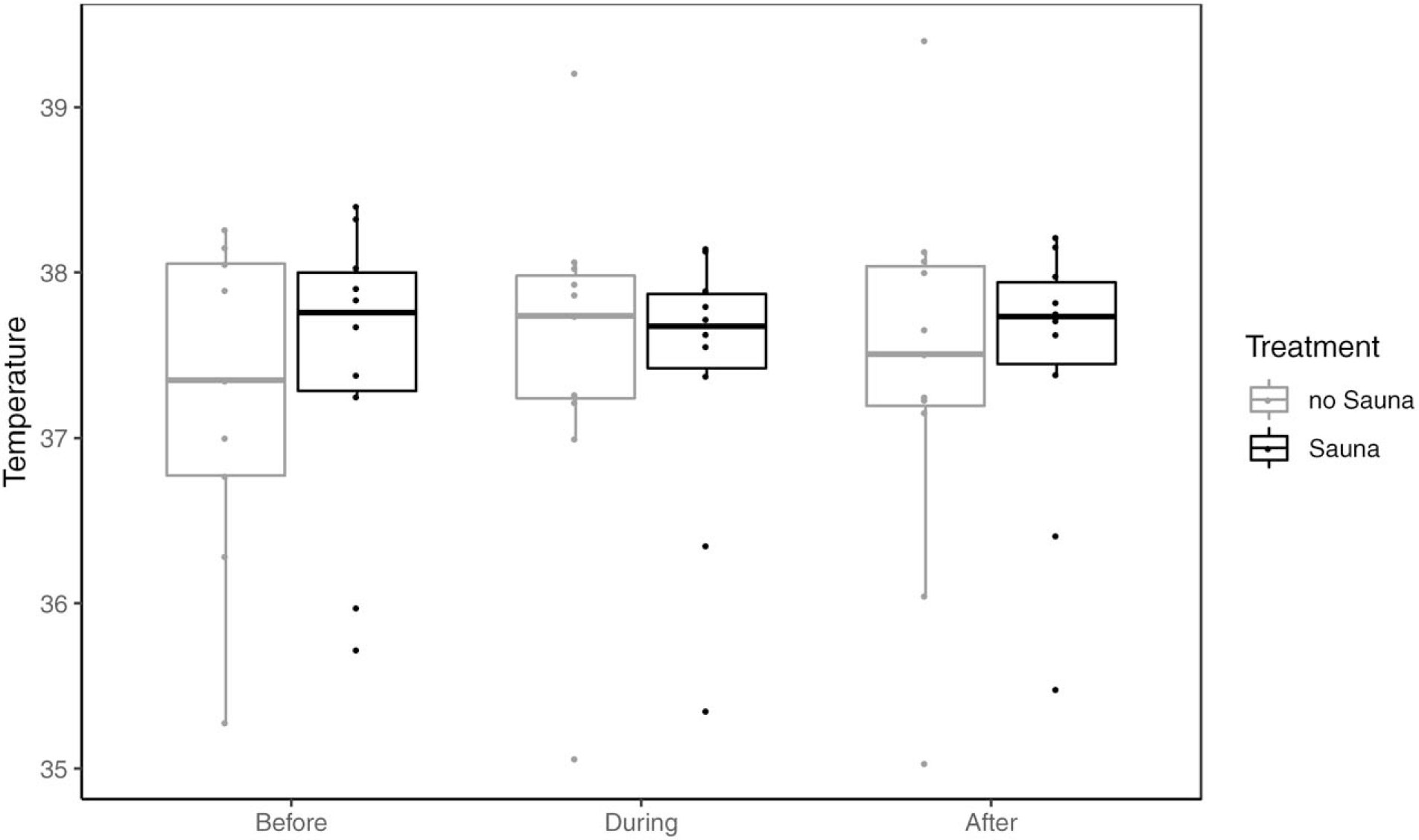
Core temperature (°C) by treatment group.

**FIGURE 4.**
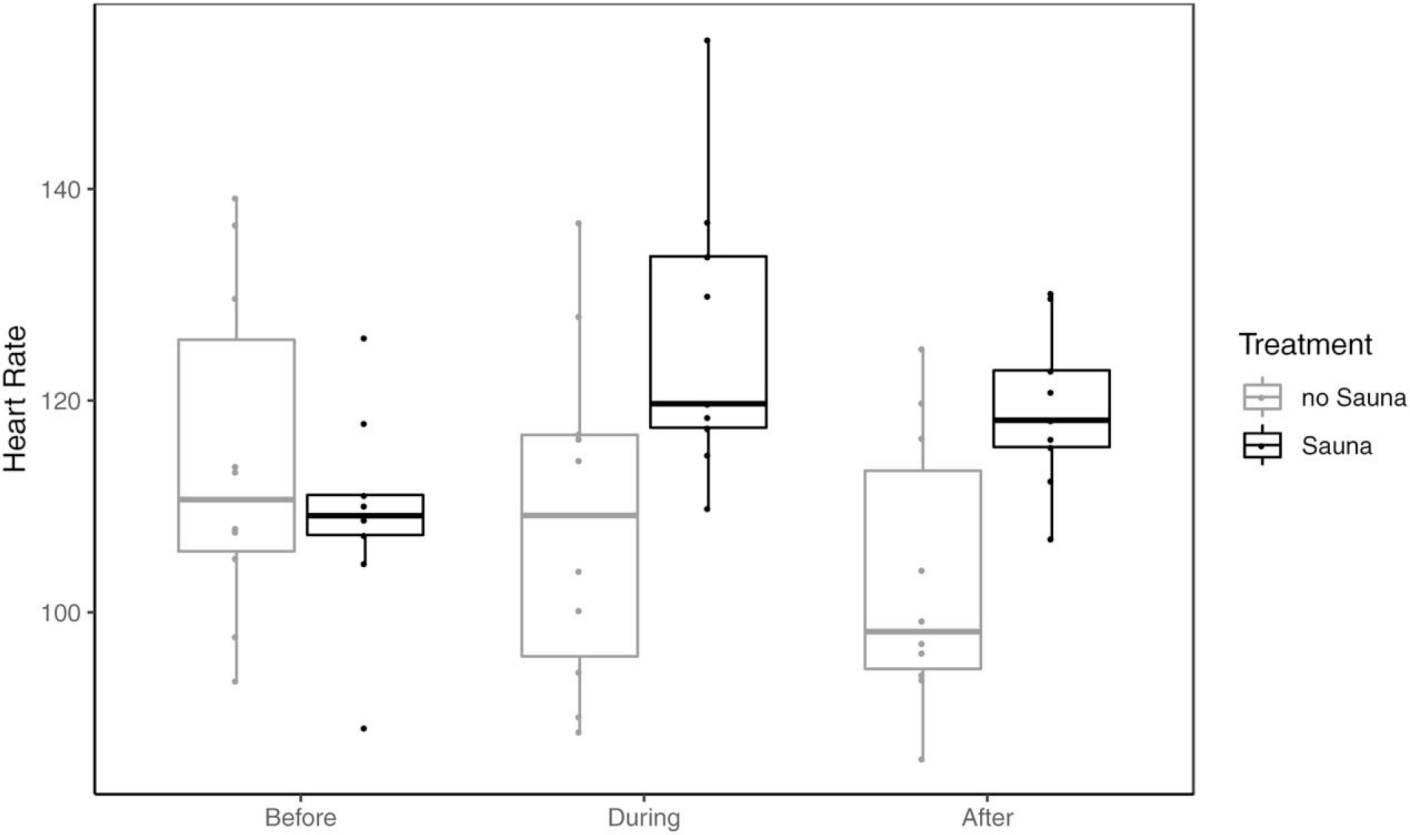
Heart rate (beats per minute) by treatment group.

There were no significant differences in mean core temperature comparing the control and sauna treatment groups during the 20-minute segments before (37.3±0.3 and 37.5±0.3°C, *P*=0.99), during (37.4±0.3°C and 37.5±0.3°C, *P*=0.95) and after (37.5±0.3 and 37.4±0.3°C, *P*=0.95) sauna treatment, respectively (Fig. 3). Mean heart rate was similar in both groups during the 20 minutes prior to sauna treatment (105±34.9 and 111±10.4 beats per minute (bpm), *P*=0.41), respectively, but compared to the control group increased in the sauna treatment group in the 20 minutes during (99.7±33.2 and 134±27.6 bpm, *P*=0.004) and after (93.1±30.6 and 126±21.5 bpm, *P*=0.006) sauna treatment, respectively (Fig. 4).

## DISCUSSION

The study results support the effectiveness of the selected fireground interventions for engineers and firefighters, and provide some initial measurements of the effects of the sauna treatment following live-fire exposure. The fireground interventions were associated with a roughly 40% reduction in urinary PAH-OHs in engineers and a slightly lower reduction in firefighters. However, no significant change was measured in captains. Sauna treatment non-significantly reduced mean urinary PAH-OHs by over 40%, with the largest reduction in urinary naphthols.

A primary fireground intervention was use of SCBA for engineers in the presence of smoke. Respiratory protection in the fire service is predominantly provided through the use of pressure demand SCBA with a full facepiece which has an assigned protection factor of over 10,000,^22^ a value supported by testing under high exertion levels in firefighters assuming reasonable facepiece fit.^23^ Firefighters generally wear SCBA where immediately dangerous to life and health concentrations of combustion products exist (the hot zone) such as during interior fire responses and ventilation. SCBA use is much less common in the warm zone where engineers operate but where combustion products from the fire can still collect.^24^ As TFD engineers had substantially less visual deposition of soot on their gear than entry teams and therefore were less likely to participate in wash down (reported in only 30% of engineers completing post-intervention surveys), the reduction in their urinary PAH-OHs is likely primarily due to increased SCBA use.

Unlike the current study, Fent et al^25^ did not observe changes in urinary PAH-OH for pump operators (engineers) when comparing samples collected pre-exposure and three hours postfire. However, their pump operator personnel had a non-significant 33% increase in benzene measured in their exhaled breath comparing postexposure to pre-exposure. Atmospheric conditions and personnel positioning relative to the fire are important factors that can contribute to an engineer’s inhalation exposure.^26^ The importance of SCBA use to prevent inhalation exposures of PAHs and other contaminants is supported by a study of training fuel packages and exposure effects on instructors and firefighters.^13^ Air purifying respirators are not recommended for conditions with potentially elevated concentrations of products of combustion, as their use during overhaul has been associated with adverse respiratory effects,^5^ and certain chemicals such as formaldehyde may break through even chemical, biological, radiological, and nuclear canisters.^27–30^

A primary fireground intervention for entry teams in the current study was wash down. Gear was cleaned using soap and water prior to doffing in order to reduce surface contamination and the potential for self-contamination as well as cross-contamination of other fire service personnel potentially coming in contact with the turnout gear, such as paramedics operating in the rehab area. Scrubbing turnout gear with dish soap and water has been shown to reduce surface PAH contamination by 85%.^18^ Naphthalene, the most volatile PAH, may penetrate the protective layers of turnout gear more than other PAHs,^31^ indicating that postfire decontamination may not prevent or minimize all potential PAH dermal exposure equally.

One unanticipated finding of the current study was the lack of effectiveness of the fireground interventions for captains. A potential explanation is increased inhalation exposure for captains in comparison to firefighters. The role of the captain at a fire scene includes radio communications between the crew and dispatchers and later the incident commander. While the firefighters gear up and don their SCBA on arrival at the scene, the captain conducts the incident size-up and radios reports to dispatchers and incoming crews. This fireground function can place the captain in the area of the working fire, resulting in a possible inhalation exposure before donning his or her SCBA. In addition, there are times when a captain removes the SCBA regulator for communication purposes as he or she exits the involved structure to communicate with the incident commander, thus exposing the captain to higher contaminant levels in comparison to the firefighters who continually use their SCBA. The unique job functions of the captain could thereby contribute to the differences observed in intervention effectiveness.

TFD practices predating the new fireground interventions included use of skin wipes and exchange of contaminated hoods on-scene. TFD personnel used soap and water or hypoallergenic alcohol and scent-free skin wipes (eg, Huggies Natural Care® Plus Wipes, Kimberly-Clark Corporation, Irving, TX) to clean off their neck, face, arms, legs and anywhere else with visible contamination both before and after the implementation period. These methods have been previously demonstrated to reduce skin PAH contamination by 54%.^18^ Laundering contaminated hoods has been shown to reduce PAH contamination by 76%.^11^

The current study survey results showed an increase in the fireground activities promoted by TFD as part of their interventions and included in their training activities prior to intervention implementation and in subsequent reminders. Additional improvement in intervention compliance would be expected to yield further reductions in fireground exposures. Organizational culture change and behavioral interventions increase the likelihood of success of programs including gear decontamination.^19,32^ However, complete compliance may not be possible, as fatigue, heat, or other factors may prevent the wash down step, and the condition of the firefighter at the time should be considered. It is also important to note that the added time on scene for postfire wash down, decontamination of equipment and bagging of gear is a likely explanation for the 10% decline in firefighters reporting showering within an hour after the response.

The sauna intervention results were equivocal with substantial but non-significant reductions in mean urinary PAH-OH Σ sums with sauna treatment compared to the control group. The standard deviation in urinary PAH-OHs was larger in the control than the sauna group. The reason for this difference is not clear, although PAH-OH from dietary sources could not be excluded as adherence with the instructions to avoid grilled meat during the study period was not confirmed. The largest reduction associated with the sauna intervention, also non-significant, was in the sum of naphthol metabolites with smaller non-significant reductions in sum of fluorenols and sum of phenanthrols. A potential explanation for this difference could be the higher volatility of naphthalene.

Little is known about the ability of heat exposure, for example, sauna, to alter the excretion of organic molecules through sweat. A study with 20 participants found that induced perspiration facilitated the excretion of PBDE congeners in sweat, although the effectiveness depended on the type of sweat-inducing intervention and the PBDE congener.^33^ A study of seven World Trade Center rescue workers evaluated the effects of the Hubbard sauna detoxification method, including multiple hours of sauna a day for at least a month, vitamin and mineral supplements and a balanced lifestyle.^34^ The study found a reduction of polychlorinated biphenyls (PCBs) in the blood of the participants while other contaminants like polychlorinated dibenzodioxins and polychlorinated dibenzofurans remained unchanged. Another study found urinary excretion of tetracycline decreased immediately after heat exposure, although the total amount of tetracycline in the 24-hour postexposure composite urine was similar to the control group,^35^ demonstrating the need for analysis of extended composite or multiple time periods of urine analysis following sauna treatment to fully measure effectiveness. If the hypothesized mechanism for sauna treatment is the release of chemicals absorbed into skin or pores, then it would also be useful in future studies to measure PAH concentrations on the skin using wipe samples after initial showering but before sauna treatment, again after sauna treatment and at similar time intervals in the control group.

A concern with any treatment, including saunas, is the potential to cause harm. The elevated heart rate seen in the current study during and after sauna treatment is an indication of heat stress. However, the foremost concern is elevated core temperature, which was not found with the current study but which has been associated in past studies of live-fire training with altered coagulation and in studies of non-firefighters with fatigue and decreased cognitive function.^36–38^ Additional firefighter sauna treatment studies are needed, potentially involving a range of sauna types, temperatures, durations and exercise conditions as well as outcome measures beyond urinary PAH-OHs, core temperature and heart rate monitoring.

This study had a number of important limitations. As the fireground interventions were not randomized, potential differences in the fires in the pre- and post-intervention periods could explain some of the reductions found in the urinary PAH-OHs. Based on the survey results, the recommended interventions were not fully implemented, suggesting that additional reductions in urinary PAH-OHs could be achieved with more complete compliance. Firefighters may have occupational exposure to PAHs that are not-fire related such as from ambient air pollution^10^ or food eaten on shift that may not be controlled by targeted interventions such as those described here. The sauna intervention study involved idealized treatment conditions which included shorter intervals between exiting the fire and entry into the sauna than could likely be achieved with actual structural fires, and it was not possible to separate the effects of the sauna itself from the additional shower taken after the sauna. The sauna intervention effect on toxicity from smoke exposure is not known. In addition, other adverse or beneficial effects not measured in the current study could potentially occur with sauna treatment.

In conclusion, the study results directly support the use of SCBA by engineers while operating at a fire incident and indirectly suggest the need for additional use of respiratory protection for other fire service personnel operating in the warm zone. The study results also support the use of wash down for entry teams, particularly as part of a broader dermal exposure reduction and contaminated gear segregation program. The infrared sauna intervention did not yield a statistically significant reduction in urinary PAH-OHs, although the number of subjects was limited. However, within the protocols developed by SFD, sauna treatment did not elevate core temperature, so there was also no clear evidence that their sauna treatment was detrimental. Further research on firefighter postexposure sauna treatment is needed.

## Supplementary Material

Supplemental Information

Table 1

## Figures and Tables

**TABLE 1.** General Characteristics of Fireground and Sauna Intervention Subjects

Intervention Groups	Fireground Baseline (*n*=242)*	Fireground Pre-intervention (*n*=104)*	Fireground Post-intervention (*n*=54)*	Sauna Intervention Control Group (*n*=12)*	Sauna Intervention Treatment Group (*n*=12)
Male *n* (%)	226 (96.6%)	98 (98.0%)	52 (100%)	11 (91.7%)	11 (91.7%)
Female *n* (%)	8 (3.4%)	2 (2.0%)	0 (0%)	1 (8.3%)	1 (8.3%)
Ethnicity and Race					
White, Non-Hispanic *n* (%)	201 (85.9%)	81 (81.0%)	43 (82.7%)	7 (63.6%)	10 (83.3%)
White, Hispanic n (%)	29 (12.4%)	18 (18.0%)	7 (13.5%)	1 (9.1%)	2 (16.7%)
Other *n* (%)	4 (1.7%)	1 (1.0%)	2 (3.9%)	3 (27.3%)	0 (0%)
Age, yrs					
Mean (SD), yrs	39.0 (8.4)	38.1 (9.1)	36.6 (8.6)	43.8 (10.7)^†^	33.3 (9.26)^†^
<30 yrs *n* (%)	37 (15.7%)	23 (23.0%)	9 (17.3%)	1 (8.33%)	5 (41.7%)
30–39 yrs *n* (%)	87 (36.9%)	36 (36.0%)	26 (50.0%)	3 (25.0%)	5 (41.7%)
≥40 yrs *n* (%)	112 (47.5%)	41 (41.0%)	17 (32.7%)	8 (66.7%)	2 (16.6%)
Smoking status *n* (%)					
Never	220 (93.6%)	95 (95.0%)	44 (84.6%)	12 (100%)	12 (100%)
Occasional	9 (3.8%)	3 (3.0%)	4 (7.7%)	0 (0%)	0 (0%)
Current	6 (2.6%)	2 (2.0%)	4 (7.7%)	0 (0%)	0 (0%)

* Total *n* for each variable may be less based on unanswered survey questions.

† *P*<0.05 by two sample *t* test comparing sauna intervention control and treatment groups.

**TABLE 2.** Fireground and Sauna Intervention Effectiveness (Geometric Means and Standard Deviations, ng/L)

	**Baseline**			**Pre-intervention**			**Post-intervention**			
**Fireground**	***n* (% ND)**	**Mean**	**SD**	***n* (% ND)**	**Mean**	**SD**	***n* (% ND)**	**Mean**	**SD**	**% Change (95% CI)** *

Σ naphthols										
Engineer	39 (0%)	6968.2	2.0	24 (0%)	14790.1	2.8	13 (0%)	8706.8	2.0	−39.6 (−64.1, 1.5)
Firefighter	82 (0%)	6840.5	3.2	57 (0%)	19235.1	2.8	30 (0%)	12235.3	2.8	**−36.4 (−58.6, −2.4)**
Captain	66 (0%)	6151.7	2.9	33 (0%)	12666.7	2.5	17 (0%)	10516.2	2.9	−10.0 (−41.2, 37.8)
Σ fluorenols										
Engineer	39 (74.4%)	198.7	1.7	24 (41.7%)	477.9	3.4	13 (38.5%)	269.8	1.7	**−65.1 (−82, −32.1)**
Firefighter	82 (67.1%)	206.4	1.7	57 (21.1%)	762.8	3.0	30 (33.3%)	459.0	3.0	**−40.7 (−60.9, −10.2)**
Captain	66 (75.8%)	195.5	1.7	33 (48.5%)	367.3	2.5	17 (41.2%)	430.9	2.9	31.8 (−16.4, 107.9)
Σ phenanthrols										
Engineer	39 (41.0%)	478.7	1.7	24 (16.7%)	1140.9	2.9	13 (30.8%)	428.7	1.5	**−68.2 (−86.7, −24.0)**
Firefighter	82 (40.2%)	481.7	1.7	56 (8.9%)	1488.1	2.6	30 (33.3%)	639.3	2.4	**−63.1 (−74.8, −46.0)**
Captain	66 (56.1%)	434.3	1.7	33 (27.3%)	955.9	2.3	17 (35.3%)	599.7	2.2	**−43.7 (−66.7, −4.8)**
Σ sums^†^										
Engineer	39 (0%)	8239.4	1.8	24 (0%)	17297.4	2.8	13 (0%)	10197.4	1.9	**−40.4 (−63.9, −2.3)**
Firefighter	82 (0%)	8396.8	2.7	57 (0%)	22706.9	2.7	30 (0%)	14454.7	2.7	**−36.2 (−56.7, −6.0)**
Captain	66 (0%)	7436.6	2.5	33 (0%)	15332.9	2.3	17 (0%)	12544.3	2.7	−11.3 (−39.4, 29.9)

**Sauna** ^‡^	**Pre-intervention**			**Post-intervention control**			**Post-intervention sauna treatment**			**% Change (95% CI)** *
		
Σ naphthols	24 (0%)	6894.6	2.4	12 (0%)	35712.2	2.7	12 (0%)	19667.7	1.7	−44.9 (−71.1, 4.8)
Σ fluorenols	24 (37.5%)	266.6	3.0	12 (8.3%)	1000.4	1.5	12 (0%)	834.1	1.7	−32.7 (−40.5, 49.0)
Σ phenanthrols	24 (8.3%)	572.9	1.8	12 (0%)	1405.9	1.4	12 (0%)	1177.2	1.6	−16.3 (−41.9, 20.6)
Σ sums^†^	24 (0%)	8495.7	2.2	12 (0%)	40012.1	2.6	12 (0%)	22604.3	1.6	−43.5 (−68.8, 2.2)

CI, confidence interval; ND, non-detectable; SD, standard deviation.

* For fireground interventions comparing pre- and post-intervention means and for sauna intervention comparing post-intervention control and sauna treatment means.

† Includes sum of Σ naphthols, Σ fluorenols, Σ phenanthrols, and 1-hydroxypyrene.

‡ All sauna intervention study values are from 12-hour composite urine samples.

**TABLE 3.** Fireground Intervention Survey Results

Status or Activity	Pre-intervention *n* (%)*	Post-intervention *n* (%)*
Turnout gear clean before the response	97 (54%)	46 (69%)
Personal protective equipment worn during fire attack:		
Turnout gear	112 (62%)	47 (70%)
Firefighting boots	110 (61%)	47 (70%)
SCBA	109 (61%)	49 (73%)
Helmet	112 (62%)	49 (73%)
Firefighting gloves	108 (60%)	47 (70%)
Eye/face protection other than SCBA	38 (21%)	23 (34%)
Personal protective equipment worn during overhaul:		
Turnout gear	48 (27%)	21 (31%)
Firefighting boots	49 (27%)	21 (31%)
SCBA	46 (26%)	21 (31%)
Helmet	48 (27%)	21 (31%)
Firefighting gloves	48 (27%)	20 (30%)
Eye/face protection other than SCBA	16 (9%)	10 (15%)
Used skin wipe or washed with water while on scene	77 (64%)	47 (78%)
Washed/rinsed/or replaced the following on scene:		
Turnout gear	12 (10%)	41 (68%)
SCBA	48 (40%)	45 (75%)
Hood	85 (71%)	44 (73%)
Bagged dirty gear before transporting it from the fire scene	5 (4%)	19 (32%)
Stowed dirty gear for transport outside of the truck cab	55 (46%)	37 (62%)
Took a full body shower within an hour after the response	69 (57%)	28 (47%)
Washed or replaced clothes within an hour after the response.	72 (60%)	30 (50%)

* The total number of responses varies based on how many subjects answered each question.

SCBA, self-contained breathing apparatus

## Learning Objectives

- Discuss the increased cancer risk in firefighters compared to the general population and the role of hydroxylated metabolites of polycyclic aromatic hydrocarbons (PAH-OHs) as biomarkers of exposure.
- Summarize the new findings on effectiveness of specific fireground interventions to reduce exposure during structural fire responses.
- Summarize the findings on the effectiveness of post-exposure infrared saunas.

## Clinical Significance:

Firefighters have a higher risk of cancer than the general population and are exposed to multiple known and suspected carcinogens including some polycyclic aromatic hydrocarbons (PAHs). Use of SCBA by engineers and wash down for entry teams, but not infrared sauna use, significantly reduce urinary PAH metabolites after fire exposures
