# Supplementary material for: Evaluation of Interventions to Reduce Firefighter Exposures
Source: J Occup Environ Med. Author manuscript; Available in PMC 2021 Dec 6. (PMC8647371; doi:10.1097/JOM.0000000000001815)
Supplement: Supplemental Information [file NIHMS1753847-supplement-Supplemental_Information.docx]

Supplemental Table 2: Individual urinary metabolites* for the sauna intervention (geometric means and standard deviations, ng/L).

|  | Baseline* | | | Post-intervention control* | | | Post-intervention sauna treatment* | | |  |
| --- | --- | --- | --- | --- | --- | --- | --- | --- | --- | --- |
| Metabolite | n (% ND) | mean | SD | n (% ND) | mean | SD | n (% ND) | mean | SD | % Change (95% CI)† |
| 1 Naphthol | 24 (25.0%) | 392.0 | 3.0 | 12 (0%) | 5638.6 | 1.9 | 12 (0%) | 3661.7 | 1.8 | -35.1 (-75.3, 70.5) |
| 2 Naphthol | 24 (0%) | 6086.0 | 3.0 | 12 (0%) | 28094.5 | 3.0 | 12 (0%) | 15534.6 | 1.8 | -44.7 (-72.6, 11.6) |
| 2 Fluorenol | 24 (45.8%) | 107.3 | 2.3 | 12 (8.3%) | 358.1 | 2.0 | 12 (0%) | 374.7 | 1.6 | 6.6 (-56.6, 162.2) |
| 3 Fluorenol | 24 (45.8%) | 90.6 | 2.0 | 12 (16.7%) | 214.6 | 2.2 | 12 (0%) | 272.8 | 1.6 | 32.7 (-36.6, 177.5) |
| 9 Fluorenol | 24 (62.5%) | 89.8 | 2.3 | 12 (8.3%) | 367.0 | 2.1 | 12 (8.3%) | 276.8 | 2.1 | -24.8 (-73.6, 114) |
| 1&3 Phenanthrol | 24 (20.8%) | 253.0 | 2.0 | 12 (0%) | 503.6 | 1.4 | 12 (0%) | 427.5 | 1.4 | -15.1 (-54.1, 57.0) |
| 2 Phenanthrol | 24 (29.2%) | 209.0 | 2.0 | 12 (0%) | 754.7 | 1.5 | 12 (0%) | 539.1 | 1.6 | -28.6 (-69.4, 66.7) |
| 4 Phenanthrol | 24 (79.2%) | 81.0 | 1.0 | 12 (16.7%) | 124.9 | 1.6 | 12 (33.3%) | 108.1 | 1.4 | -18.4 (-44.5, 20.2) |
| 1 Hydroxypyrene | 24 (54.2%) | 222.5 | 2.8 | 12 (33.3%) | 392.3 | 3.0 | 12 (16.7%) | 426.9 | 2.6 | 26.0 (-66.6, 375) |

* From 12-hour composite samples; ND = non-detectable; SD = standard deviation; CI = confidence interval; † Comparing post-intervention control and sauna treatment groups.
